# Comparison of MRI-derived pulmonary edema measures with LVEDP and serum BNP

**DOI:** 10.1186/1532-429X-11-S1-P41

**Published:** 2009-01-28

**Authors:** Kelvin Chow, Mustafa Toma, Ben Esch, Jessica Scott, Mark Haykowsky, Richard Thompson, Ian Paterson

**Affiliations:** 1 University of Alberta, Edmonton, AB Canada; 2 University of British Columbia, Vancouver, BC Canada

**Keywords:** Heart Failure Patient, Cardiogenic Pulmonary Edema, Heart Failure Group, Percentage Water Content, Lung Water Content

## Introduction

Cardiogenic pulmonary edema is the accumulation of fluid in the interstitial and often alveolar space of the lungs, caused by increased hydrostatic pressure leading to extravasation of fluid. Currently, assessment of edema is limited to qualitative chest x-ray and occasionally CT. It is known that MRI can measure lung water content [1], but systematic sources of error such as B_1_ field heterogeneity and breath-hold variability have not previously been accounted for, and MRI-derived lung water has not been compared with invasively measured heart pressures.

## Purpose

To compare lung water content measured using free-breathing MRI in populations of healthy volunteers and heart failure patients to left ventricular end-diastolic pressure (LVEDP) and blood serum b-type natriuretic peptide (BNP) concentration.

## Methods

7 healthy male volunteers (31 ± 9 yrs) and 10 heart failure patients (53 ± 14 yrs; 8 male) were imaged on a Siemens Sonata 1.5 T MRI scanner with informed consent and IRB approval. A half-Fourier single-shot turbo spin-echo (HASTE) pulse sequence was used with typical parameters: excitation flip angles 60°/120°, 1.4 × 1.4 × 8.0 mm resolution, ECG gating (end-diastolic imaging), 12 ms effective TE, >5 s TR, 7 repetitions during free breathing, 6 minute total acquisition time for 10 sagittal slices across both lungs.

An automated image morphing algorithm was used to deform each image to a reference respiratory phase (end expiration) and signal intensity corrections were applied to account for normal changes during respiration. The lung was manually traced once per sagittal slice and an automated algorithm segmented the lung into smaller regions (~3.5 mm^2^). Bright blood vessel contributions were removed with regional thresholding.

Images were acquired with two different flip angles and a modified double angle method [2] was used to measure and correct for a heterogeneous B_1_ radiofrequency field. A large region of the liver, visible in a mid-sagittal slice of the right lung, was used as a source of known water density (~70%). For each heart failure patient, LVEDP was measured with an invasive catheter immediately before the MRI scan and BNP levels were collected immediately after.

## Results

**Figure 1:**
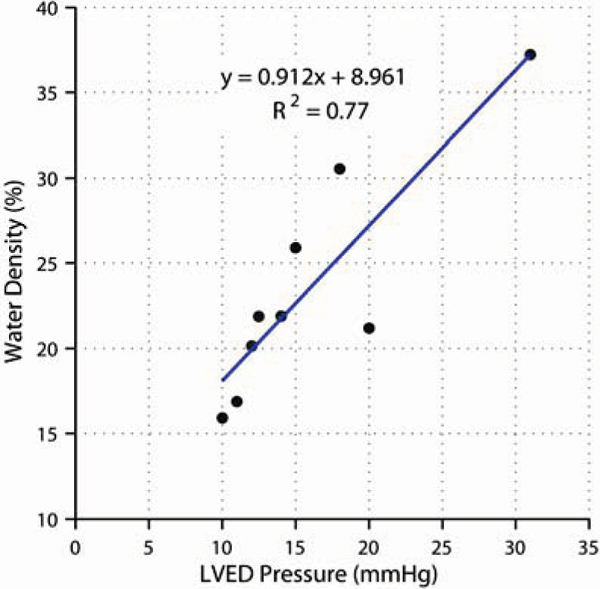
**MRI-derived lung water as a function of LVEDP in heart failure**.

**Figure 2:**
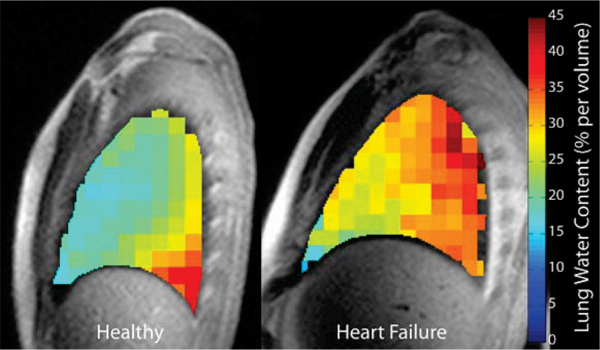
**MRI-derived lung water (mid-sagittal right lung) for a healthy subject (total average lung water 21.7%) and a heart failure patient (37.2%)**.

Average lung water content of lungs in the healthy population was 22.5 ± 2.4% (percentage water content per unit volume), similar to previous reports [3]. Lung water content in the heart failure group is considerably more variable, ranging from 16% to 37%, with good correlation to both LVEDP (R^2^ = 0.77, Fig. 1) and BNP concentrations (R^2^ = 0.75). LVEDP and BNP also correlated well (R^2^ = 0.57). Lung water maps for a mid-sagittal slice through the right lung are shown for both a healthy individual and a heart transplant patient (Fig. 2).

For a prescribed 90° tip angle, the computed B_1_ field maps indicate that the average flip angle achieved within the left lung is 77.2 ± 2.3° and only 61.1 ± 1.8° in the right lung. Correcting for this B_1_ heterogeneity resulted in an absolute increase in lung water density in the right lung of 9.6 ± 1.5%, with negligible correction in the left lung.

## Conclusion

A free-breathing MRI approach to quantifying pulmonary edema incorporating corrections for heterogeneous B_1_ fields was implemented using a conventional HASTE sequence. Lung water density was found to be tightly grouped within a population of healthy individuals, with good correlation to both gold standard LVEDP and BNP concentrations in heart failure patients. MRI-derived lung water, measured as part of comprehensive cardiovascular exam, is thus a potential surrogate for LVEDP.
